# Effects of end-stage osteoarthritis on markers of skeletal muscle Long INterspersed Element-1 activity

**DOI:** 10.1186/s13104-022-06113-0

**Published:** 2022-07-07

**Authors:** Shelby C. Osburn, Matthew A. Romero, Paul A. Roberson, Petey W. Mumford, Derek A. Wiggins, Jeremy S. McAdam, Devin J. Drummer, S. Louis Bridges, Marcas M. Bamman, Michael D. Roberts

**Affiliations:** 1 School of Kinesiology, Auburn University, 301 Wire Road, Office 260, Auburn, AL 36849 USA; 2 Department of Microbiology, Immunology, and Molecular Genetics, University of California, Los Angeles, CA USA; 3 Department of Cellular and Molecular Physiology, Pennsylvania State University College of Medicine, Hershey, PA USA; 4 School of Health Sciences, Lindenwood University, Saint Charles, MO USA; 5 Department of Cell, Developmental, and Integrative Biology, University of Alabama at Birmingham, Birmingham, AL USA; 6 UAB Center for Exercise Medicine, University of Alabama at Birmingham, Birmingham, AL USA; 7 Department of Medicine, Hospital for Special Surgery, New York, NY USA; 8 Division of Rheumatology, Weill Cornell Medical Center, New York, NY USA; 9 Florida Institute for Human and Machine Cognition, Pensacola, FL USA

**Keywords:** Osteoarthritis, Skeletal muscle, LINE-1, STING

## Abstract

**Objective:**

Long INterspersed Element-1 (L1) is an autonomous transposable element in the genome. L1 transcripts that are not reverse transcribed back into the genome can accumulate in the cytoplasm and activate an inflammatory response via the cyclic GMP-AMP (cGAS)-STING pathway. We examined skeletal muscle L1 markers as well as STING protein levels in 10 older individuals (63 ± 11 y, BMI = 30.2 ± 6.8 kg/m^2^) with end-stage osteoarthritis (OA) undergoing total hip (THA, n = 4) or knee (TKA, n = 6) arthroplasty versus 10 young, healthy comparators (Y, 22 ± 2 y, BMI = 23.2 ± 2.5 kg/m^2^). For OA, muscle was collected from surgical (SX) and contralateral (CTL) sides whereas single vastus lateralis samples were collected from Y.

**Results:**

L1 mRNA was higher in CTL and SX compared to Y (p < 0.001 and p = 0.001, respectively). Protein expression was higher in SX versus Y for ORF1p (p = 0.002) and STING (p = 0.022). While these data are preliminary due to limited n-sizes and the lack of a BMI-matched younger control group, higher L1 mRNA expression, ORF1p and STING protein are evident in older versus younger adults. More research is needed to determine whether cGAS-STING signaling contributes to heightened muscle inflammation during aging and/or OA.

**Supplementary Information:**

The online version contains supplementary material available at 10.1186/s13104-022-06113-0.

## Introduction

Long INterspersed Element-1 (LINE-1 or L1) is the only autonomous retrotransposon in the human genome and is able to “copy and paste” itself via an RNA intermediate [1]. Given this gene’s ability undergo retrotransposition, it makes up ~ 17% of the human genome [2]. A full length, active L1 transcript contains a 5’ untranslated region (UTR), two open reading frames (ORF1 and ORF2), and a 3’ UTR with a weak polyadenylation signal and poly(A) tail [3]. During the process of retrotransposition, the 6 kilobase gene is transcribed and the transcript is exported to the cytoplasm where the two ORFs are translated into proteins with unique functions that show a ‘cis-preference’ for the mRNA from which they were translated [4]. ORF1 and ORF2 bind to the L1 transcript to form a L1 ribonucleoprotein (RNP) that has the necessary machinery to localize back to the nucleus [5]. ORF1p is an RNA binding protein with nuclear localization activity [6] that binds to L1 mRNA in trimers along the transcript [7]. ORF2p contains both endonuclease (EN) and reverse transcriptase (RT) domains [8, 9] and binds to the L1 transcript towards the 3’ end [10]. There are two heavily researched mechanisms involved in regulation of L1 expression including hypermethylation of the cytosine residues in the internal promoter and histone modification via deacetylases [11, 12]. In this regard, it is generally recognized that L1 methylation is a strong signal for transcription inhibition. In fact, approximately 13% of the L1 promoter region contains these cytosine residues and it can exhibit a methylation state anywhere from 20 to 100%.

**Fig. 1:**
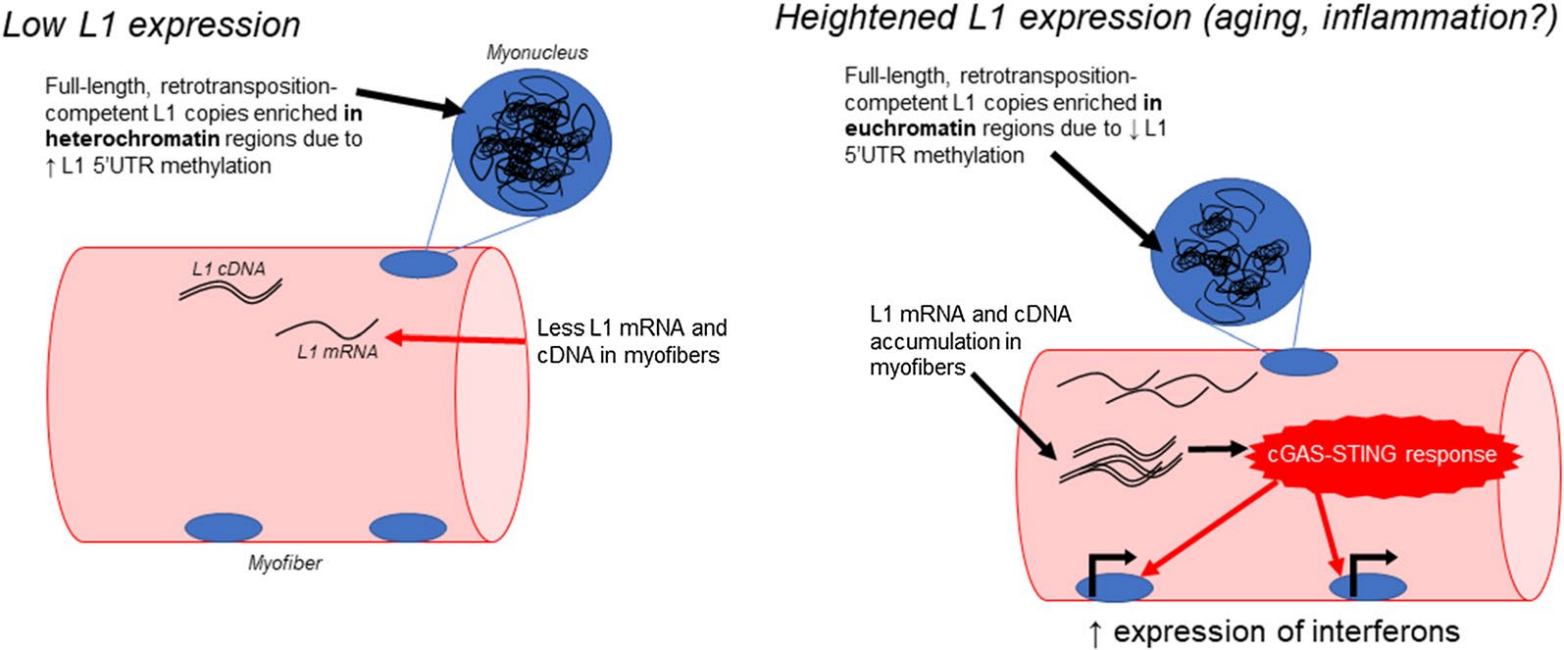
Schematic of L1 expression and the involvement of cGAS-STING. This schematic illustrates how increased L1 expression may initiate inflammatory signaling through cGAS-STING based on prior literature, and this model was the basis for the current study

It has been posited that the L1 RNP needs a break in the nuclear envelope to return to the nucleus in order to undergo retrotransposition [13]. This poses an interesting problem for skeletal muscle since muscle cells are post-mitotic and do not undergo cellular division to disrupt the nuclear envelope. Simon and colleagues have shown that an accumulation of L1 cDNA copies in the cytoplasm can induce an inflammatory response through the cyclic GMP-AMP (cGAS) DNA sensing pathway [14, 15]. Bai and Liu go into great detail regarding the cGAS pathway and its implications [16]. Briefly, the cGAS-GMP-AMP receptor stimulator of interferon genes (STING) signaling cascade begins with the sensing and binding of dsDNA to cGAS to activate it. Once activated, cGAS catalyzes the formation of 2′3′-cGAMP, which then activates STING proteins. Subsequently, STING can translocate and recruit TBK1, setting off a cascade that results in type I interferon production. Since L1 is not able to return to the nucleus efficiently, L1 mRNA or cDNA accumulation in the sarcoplasm of skeletal muscle may induce this inflammatory response. Figure 1 summarizes these processes.

Osteoarthritis (OA) is the most prevalent joint disorder in aged populations, as it affects a majority of persons over the age of 65 years of age [17]. Beyond affecting joints via cartilage degeneration and elevated inflammation, OA can also affect surrounding skeletal muscle. For instance, knee OA has been shown to elevate the expression of proinflammatory cytokines as well as inflammatory signaling mediators in the vastus lateralis muscle [18]. However, there is a paucity of evidence regarding how OA mechanistically causes muscle inflammation [19]. Moreover, it is unknown how skeletal muscle L1 activity is affected with OA-induced inflammation. Given the link between L1 signaling and inflammation through cGAS-STING signaling, the purpose of this preliminary report was to determine whether these L1 markers or STING protein levels were elevated in individuals with pathological inflammation (i.e., those with end-stage OA) when compared to a younger, apparently healthy cohort.

## Main text

### Methods

#### Experimental design

Skeletal muscle biopsies were obtained perioperatively from OA patients (63 ± 11 years old) undergoing total hip or total knee arthroplasty (THA n = 4; TKA n = 6; total n = 10) enrolled in the TWEAK Trial (R01HD084124, NCT02628795) at the University of Alabama at Birmingham (Birmingham, AL, USA). Notably, these participants were analyzed from a larger trial and more information on the participants can be found elsewhere [20]. Muscle samples from the surgical limb (SX) for TKA were collected from the vastus medialis and for THA from the gluteus maximus (posterior THA approach) or tensor fascia latae (anterior THA approach). Muscle from the contralateral (CTL) thigh was collected from the vastus lateralis (VL), providing a within-subject control. Young, healthy individuals (Y; 22 ± 2 years old) (IRB protocol #: 18–266 AR 1806; Auburn, AL, USA) served as a comparator group (denoted as “Y”, n = 10) with muscle samples collected from VL. L1 mRNA expression and DNA content were quantified using primer sets for ORF1. DNA methylation status and chromatin state of the L1 promoter were also interrogated. Protein targets included ORF1p and STING. Below provides abbreviated methods for each of these experiments, and more details regarding muscle biopsies can be found elsewhere [21].

#### Biochemical analyses

Muscle samples were removed from – 80 °C storage, tissue was crushed on a liquid nitrogen-cooled ceramic mortar and pestle, and approximately 10 mg of muscle from each participant were used to isolate RNA via the Qiagen RNeasy kit (Qiagen, Venlo, Netherlands) per the manufacturer’s recommendations. qPCR techniques as well as primers used can be found in our recent report by Roberson et al. [21]. The geometric mean of two housekeeping genes (PPIA, FBL) was used to normalize mRNA expression results. Notably, n = 7 older participants and n = 9 younger participants were assayed for this marker due to very high variability in the normalized values. Outliers were determined using the 2 standard deviation method.

Approximately 10 mg of frozen muscle tissue were also processed using the commercially available DNeasy Blood & Tissue Kit (QIAGEN, Venlo, Netherlands) per the manufacturer’s recommendations including RNase treatment. DNA was eluted with 100 μL of elution buffer from the kit per manufacturer’s recommendations, and DNA concentrations were determined in duplicate at an absorbance of 260 nm by using a NanoDrop Lite (Thermo Scientific, Waltham, MA, USA). L1 promoter methylation analysis was performed on isolated DNA using a commercially available methylated DNA immunoprecipitation (MeDIP) kit (Abcam, Cambridge, MA, USA). L1 chromatin accessibility was assessed from each sample using a commercially available kit (Chromatin Accessibility Assay Kit, product #: ab185901; Abcam) per the manufacturer’s recommendations. Finally, L1 gDNA expression was quantified using qPCR. All these methods have been extensively described in our recent report by Roberson et al. [21]. Notably, n = 6–8 older participants and n = 8–10 younger participants were assayed due to tissue limitations.

For western blot analysis, approximately 15 mg of frozen muscle were placed in 1.7 mL microcentrifuge tube containing 500 μL of ice-cold cell lysis buffer [20 mM Tris–HCl (pH 7.5), 150 mM NaCl, 1 mM Na-EDTA, 1 mM EGTA, 1% Triton, 20 mM sodium pyrophosphate, 25 mM sodium fluoride, 1 mM β-glycerophosphate, 1 mM Na3VO4 and 1 μg/mL leupeptin] (Cell Signaling; Danvers, MA, USA). Tissues were homogenized, prepared for western blotting, and subjected to electrophoresis techniques as described elsewhere [21]. Membranes were then blocked at room temperature with 5% nonfat milk powder in Tris-buffered saline with 0.1% Tween-20 (TBST) for one hour. The following primary antibodies were incubated overnight at 4ºC in a solution of TBST containing 5% bovine serum albumin (BSA; Ameresco): Mouse anti-ORF1p (1:1,000; Abcam, catalog no. ab76726), Rabbit anti-STING (1:1000; CST, catalog no. 50494 T). The following day, membranes were incubated with HRP-conjugated anti-mouse or anti-rabbit IgG secondary antibodies (1:2000; Cell Signaling cat# 7076 and 7074, respectively) in a solution of TBST containing 5% BSA at room temperature for one hour. Thereafter, membranes were developed using an enhanced chemiluminescent reagent (Luminata Forte HRP substrate; EMD Millipore, Billerica, MA, USA) with band densitometry assessed by use of a digital gel documentation system and associated densitometry software (ChemiDoc; Bio-Rad Laboratories, Hercules, CA, USA). Densitometry on white band values for each target was normalized to a corresponding dark band using Ponceau densitometry values. Additionally, values were normalized to the Y group to yield relative protein expression levels. We opted against assaying ORF2p protein herein given that others have used mass spectrometry and antibody-based methods to show that ORF2p protein levels are not detected in human cell lines [22].

#### Statistical analysis

Statistics were performed using SPSS v. 23 (IBM, Armonk, NY, USA). Tissue markers were checked for normality using the Shapiro–Wilk test. Normally distributed data was analyzed using one-way ANOVAs, and if statistical significance was obtained (p < 0.05), then a Fisher’s LSD post hoc tests were performed. Non-normally distributed data was analyzed using the Kruskal–Wallis test, and if statistical significance was obtained, then a Mann–Whitney U post hoc tests were performed. For discussion purposes, some markers were analyzed using forced post hoc tests. All data are presented as mean ± standard deviation values.

### Results

#### Participant characteristics

Participant characteristics from the younger (n = 10; 3 men, 7 women) and OA (n = 10; 2 men, 8 women) participants were as follows: age, 23 ± 2 years old versus 63 ± 11 years old; body mass index, 23.2 ± 2.5 kg/m^2^ versus 30.2 ± 6.8 kg/m^2^. OA participants possessed significantly greater values for age and BMI (p < 0.05).

#### ORF1 mRNA and DNA content, L1 promoter DNA methylation, and L1 chromatin accessibility

**Fig. 2:**
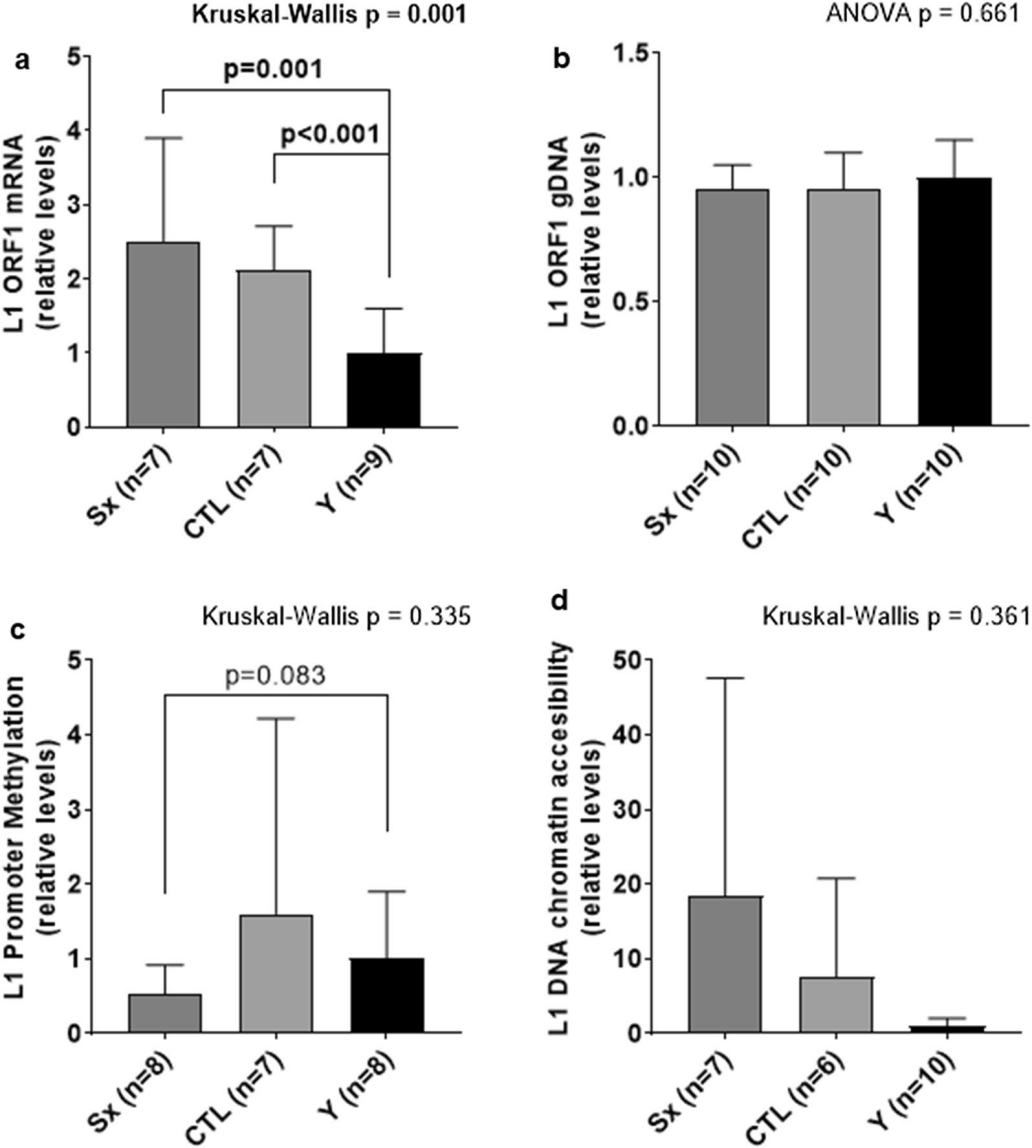
L1 mRNA expression is higher in older participants regardless of limb. ORF1 mRNA expression (panel **a**) and gDNA content (panel **b**), L1 Promoter methylation status (panel **c**), and L1 chromatin accessibility (panel **d**). All variables in OA patients are presented in the surgical (SX) and control (CTL) thighs as fold-change from the young comparator group (Y). Different letters above bars indicate a significant difference between groups (p < 0.05), and all data are presented as mean ± standard deviation values

ORF1 mRNA was significantly higher in the SX and CTL leg of the OA participants compared to the Y control (p = 0.001 and p < 0.001, respectively; Fig. 2a). However, there were no differences in gDNA content for ORF1 in any of the groups (Fig. 2b), or the chromatin accessibility of the L1 promoter (Fig. 1d). There was no difference in L1 promoter methylation state (p = 0.335), but with a forced t-test, SX was trending to be in a more hypermethylated state compared to the Y control (p = 0.083; Fig. 2c).

#### Protein expression of select targets

**Fig. 3:**
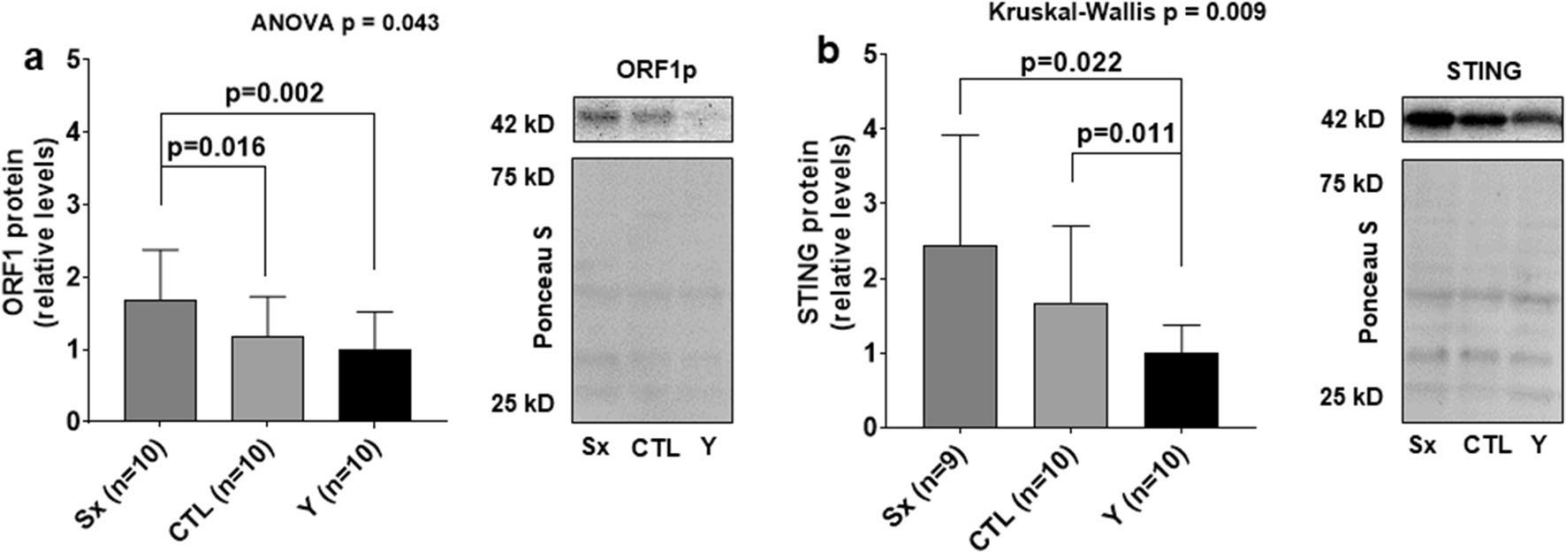
ORF1p and STING protein levels are higher in older participants, and ORF1p is higher in SX versus CTL in older participants. ORF1p expression with representative image (panel **a**) and STING protein expression with representative image (panel **b**). All variables in OA patients are presented in the surgical (SX) and control (CTL) thighs as fold-change from the young comparator group (Y). Different letters above bars indicate a significant difference between groups (p < 0.05), and all data are presented as mean ± standard deviation values. Notably, the right inset images are representative of one older participant (SX and CTL leg) and a younger participant. Ponceau S images are also provided to show protein loading did not differ between lanes

ORF1p expression was higher in SX compared to the Y control (p = 0.002) and in SX compared to CTL (p = 0.016), but there were no differences between Y and CTL (Fig. 3a). STING protein expression was also higher in SX compared to Y (p = 0.022) and CTL compared to Y (p = 0.011), but there were no differences between SX and CTL legs (Fig. 3b). Full-length blots/gels are presented in Additional file 1: Fig. S1.

### Discussion

We sought to examine if skeletal muscle L1 markers and STING protein levels were elevated in participants with end-stage OA compared to a group of young, healthy individuals. Notably, we observed higher ORF1 mRNA expression in the OA participants, regardless of leg. Additionally, we observed higher protein expression of ORF1p and STING proteins in the surgical leg of OA vs. contralateral. These findings suggest heightened L1 and cGAS-STING signaling in the affected leg of OA patients may coincide with or even partially drive increased inflammation.

We and others have previously reported skeletal muscle L1 mRNA expression increases with aging in rodents [23, 24] and humans [21]. The results of this study align with those findings; specifically, regardless of limb, L1 mRNA expression markers were elevated in older versus younger participants. However, this was not seemingly due to alterations in L1 methylation and/or chromatin state. Rather, greater L1 levels in older humans herein may have been due to other transcriptional mechanisms that were not evaluated (e.g., increased transcription factor binding to the L1 promoter). In this regard, more research is needed to further elucidate how aging affects L1 regulatory mechanisms.

Another novel finding from this study includes the elevated ORF1p protein levels in the surgical leg. While these findings are preliminary, this suggests OA either increases the translation of L1 mRNA or increases the protein stability of ORF1p. The upregulation of L1 markers as well as STING protein levels in the surgical leg of older patients also supports the notion that L1 transcript or cDNA accumulation in the sarcoplasm may stimulate the cGAS-STING pathway. Alternatively stated, a heightened inflammatory response in the surgical leg may be due to L1-stimulated cGAS-STING signaling. These data agree in principle with rodent data by Lucchinetti et al. [25] that suggests ischemic injury to cardiac muscle, which leads to an inflammatory cascade [26], increases L1 pathway activity in cardiac tissue. However, again, future research is needed to determine the relationship between increased L1 signaling in inflammation-prone muscle, and whether this mechanistically drives (rather than coincides with) the inflammatory process. Moreover, it may be possible that the combinatorial effects of inflammation and disuse of the SX limb, rather than inflammation alone, acted to affect these markers. Hence, determining how disuse affects skeletal muscle L1 markers is also warranted.

#### Limitations

There are various limitations to this study that need to be considered. First, due to the nature of the TWEAK trial, the n-size was limited for our analyses. Additionally, tissue limitations from muscle biopsies led to a reduced n-size for certain markers. Furthermore, the muscle that was biopsied differed depending on the group. This could potentially lead to differences in expression and regulation that needs to be addressed with further research. Also notable is the lack of a BMI-matched control group. Given that older participants were obese, and younger participants presented normal BMI values, this convolutes interpretations regarding whether aging or body mass affected the assayed markers more. Lastly, our interrogation of the cGAS DNA sensing pathway only included the STING protein marker. This is an important marker because it is the modulator of the pathway, but quantification of downstream inflammatory response markers would strengthen our findings. Regardless of limitations, this study was a preliminary investigation that resulted in novel findings that could impact OA disease progression and rehabilitation.

## Supplementary Information

**Additional file 1:** Embedded images are raw Western blot images for assayed targets.

## Data Availability

All raw data can be obtained by emailing the corresponding author (mdr0024@auburn.edu).

## Abbreviations

cGAS: Cyclic GMP-AMP
CTL: Contralateral
EN: Endonuclease
LINE-1 or L1: Long INterspersed Element-1
MeDIP: Methylated DNA immunoprecipitation
OA: Osteoarthritis
ORF: Open reading frame
RNP: Ribonucleoprotein
RT: Reverse transcriptase
STING: Stimulator of interferon genes
SX: Surgical
TBST: Tris-buffered saline with 0.1% Tween-20
THA: Total hip arthroplasty
TKA: Total knee arthroplasty
UTR: Untranslated region
VL: Vastus lateralis
Y: Young control
