# Supplementary material for: Effects of end-stage osteoarthritis on markers of skeletal muscle Long INterspersed Element-1 activity
Source: BMC Res Notes. 2022 Jul 7;15:245. doi: 10.1186/s13104-022-06113-0 (PMC9264706; doi:10.1186/s13104-022-06113-0)

ORF1p Ponceau gel1


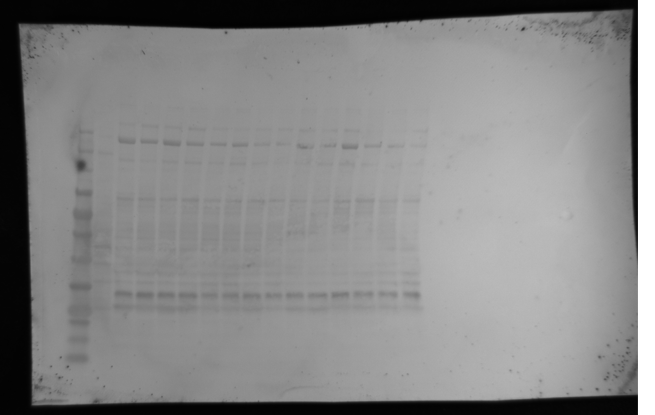


ORF1p Image gel 1


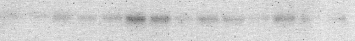


ORF1p Ponceau gel 2


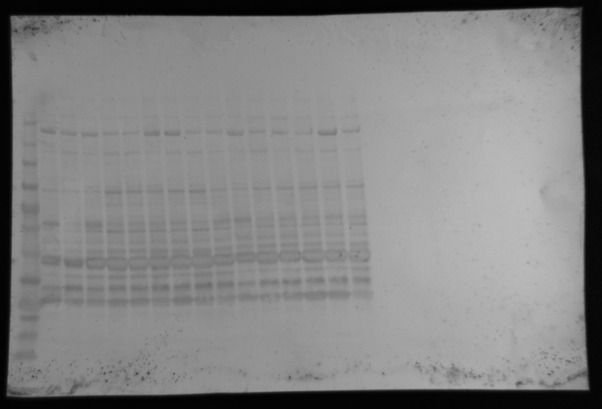


ORF1p Image gel 2


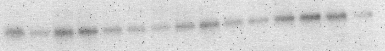


STING Ponceau gel 1


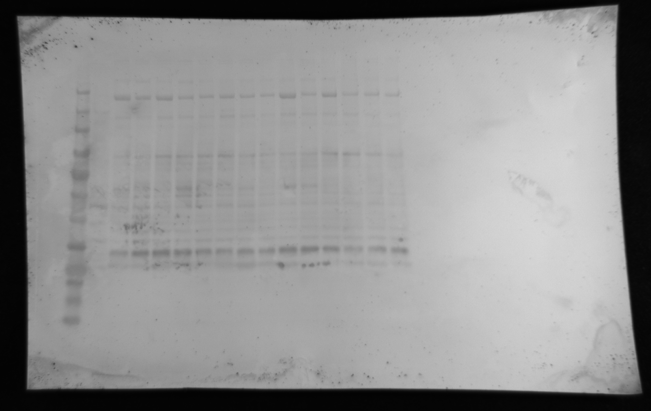


STING Image gel 1


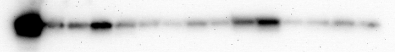


STING Ponceau gel 2


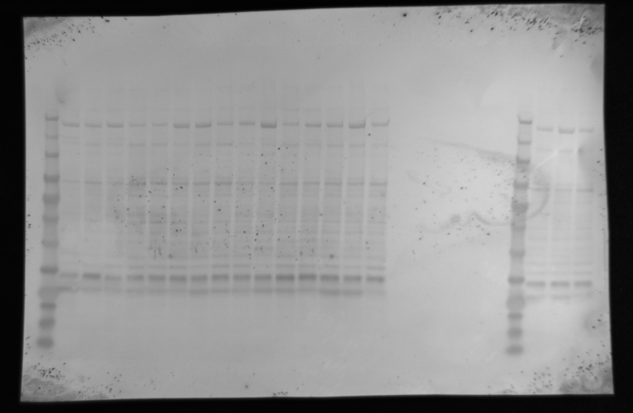


STING Image gel 2


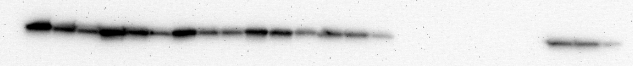

Supplement: Supplementary file 1 — Additional file 1: Embedded images are raw Western blot images for assayed targets. [file 13104_2022_6113_MOESM1_ESM.docx]
